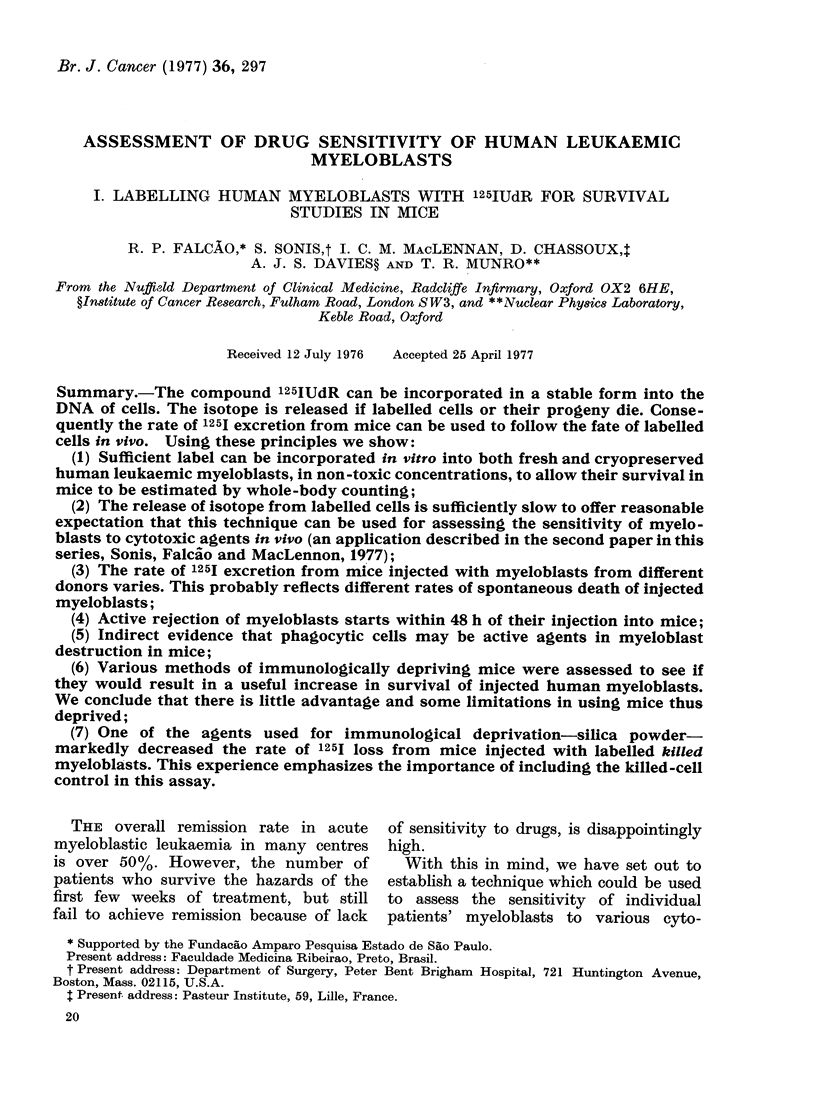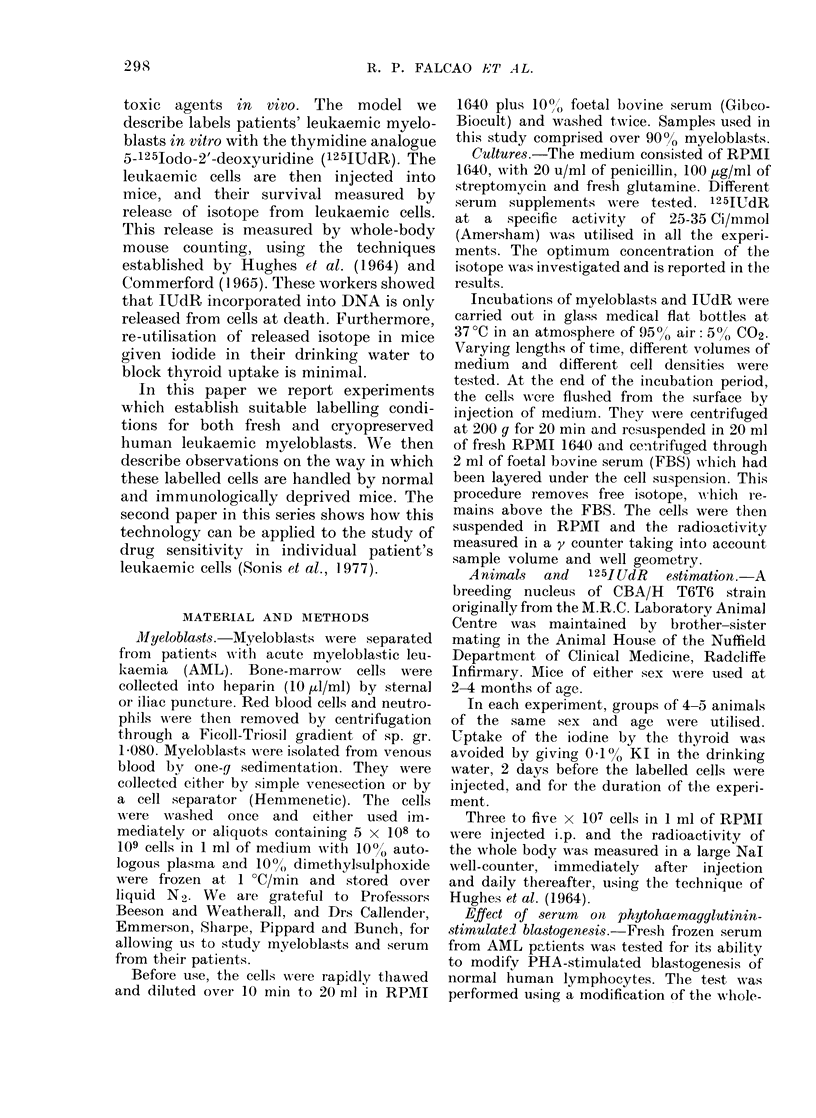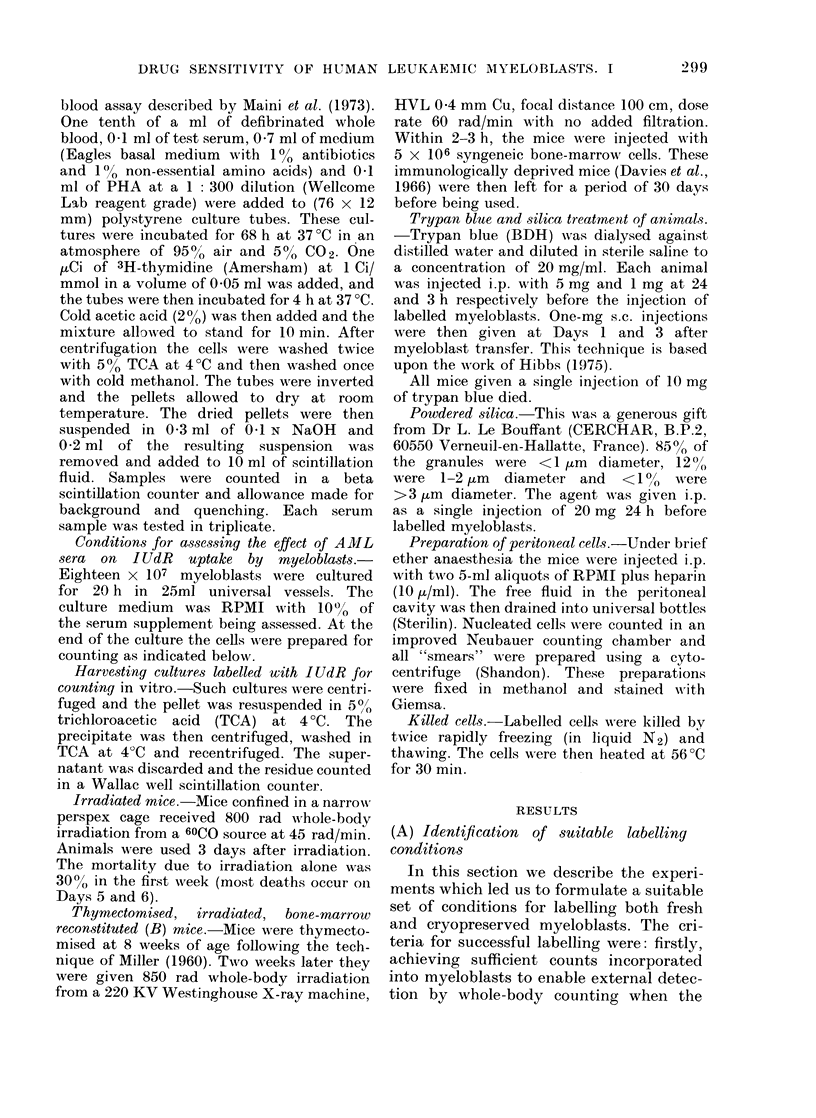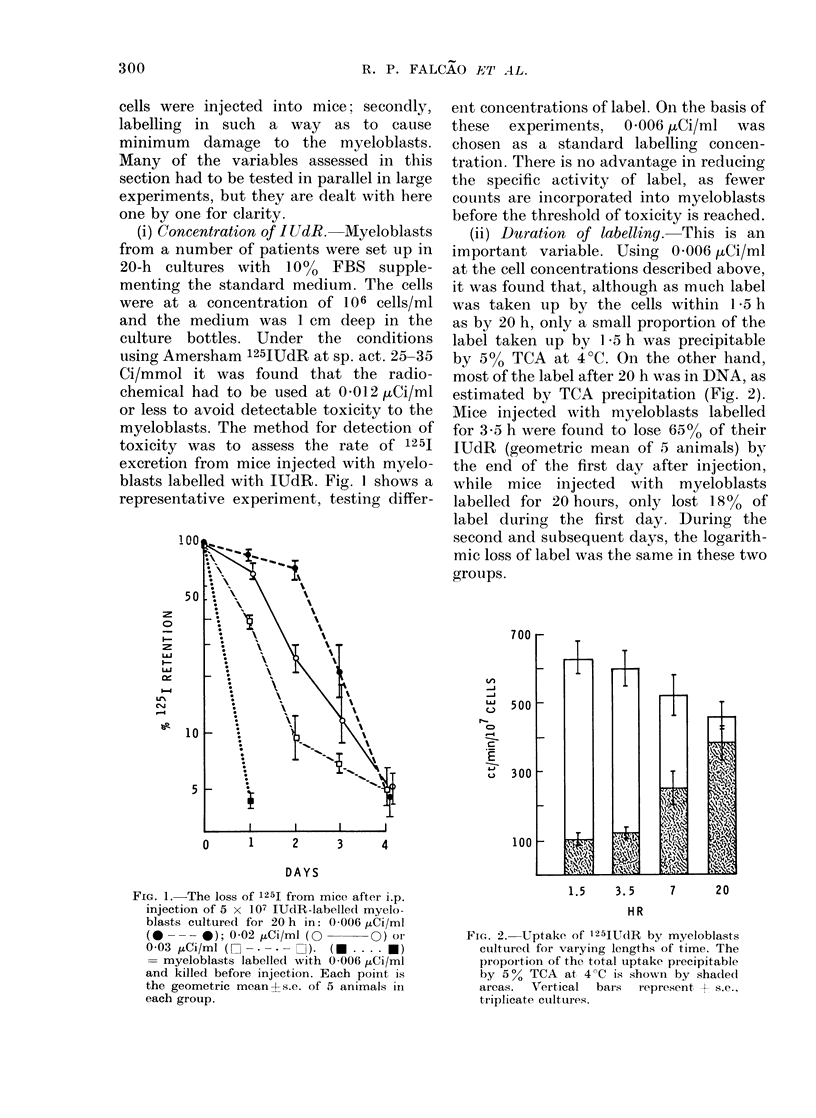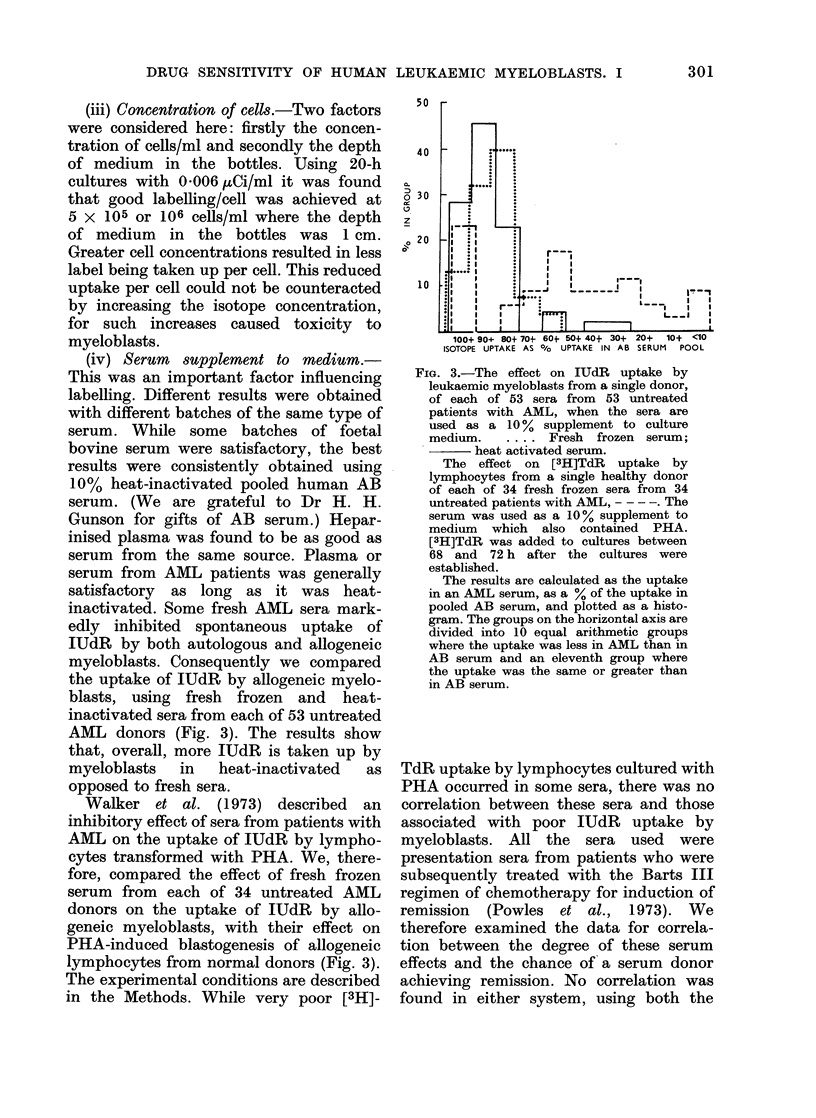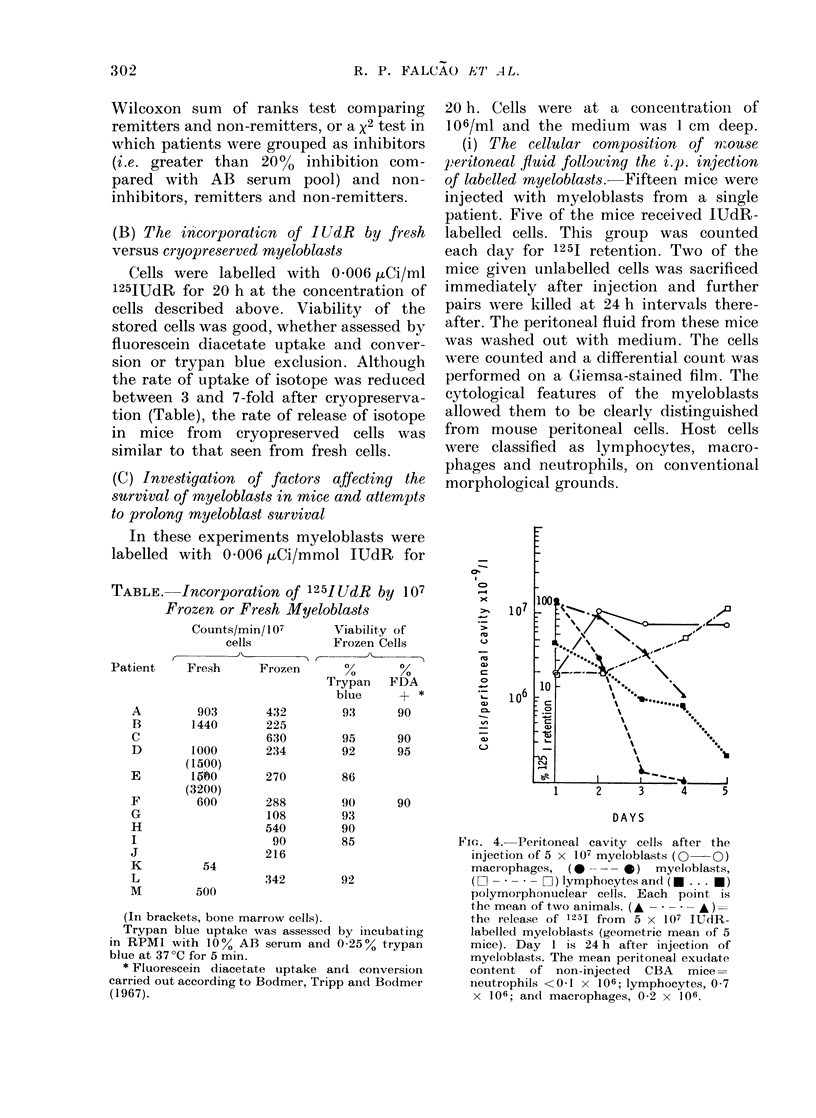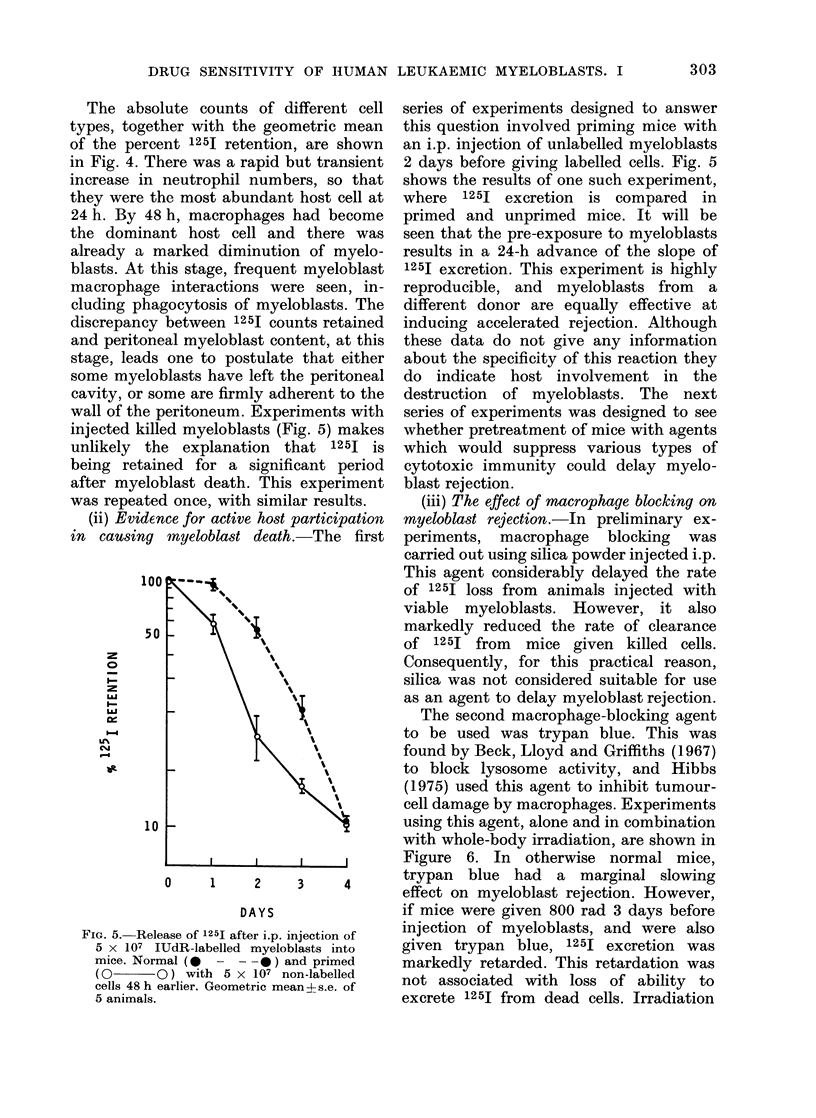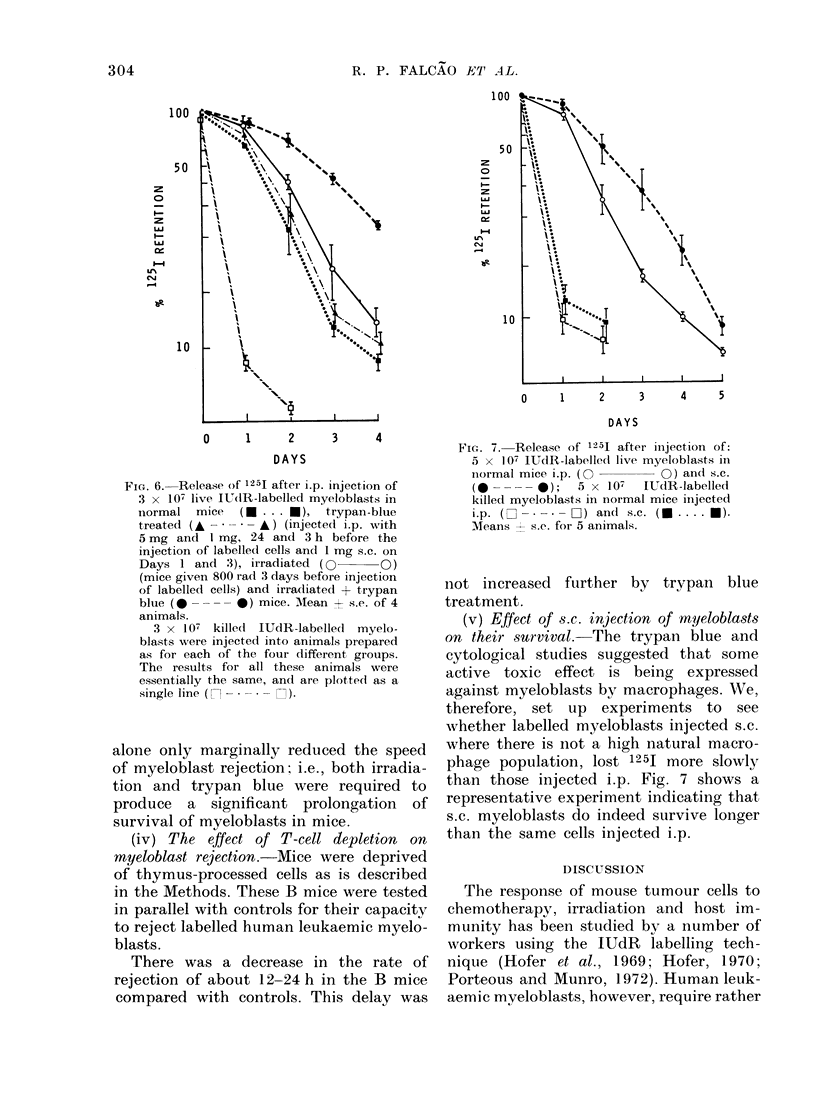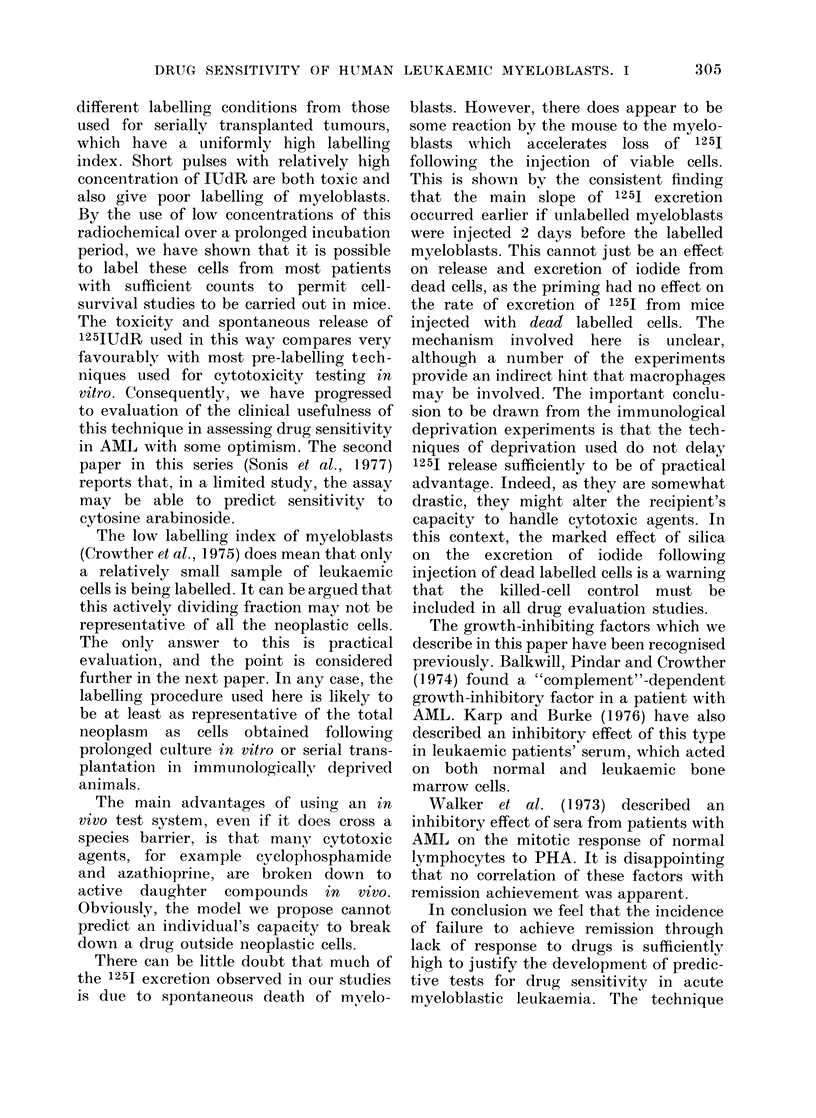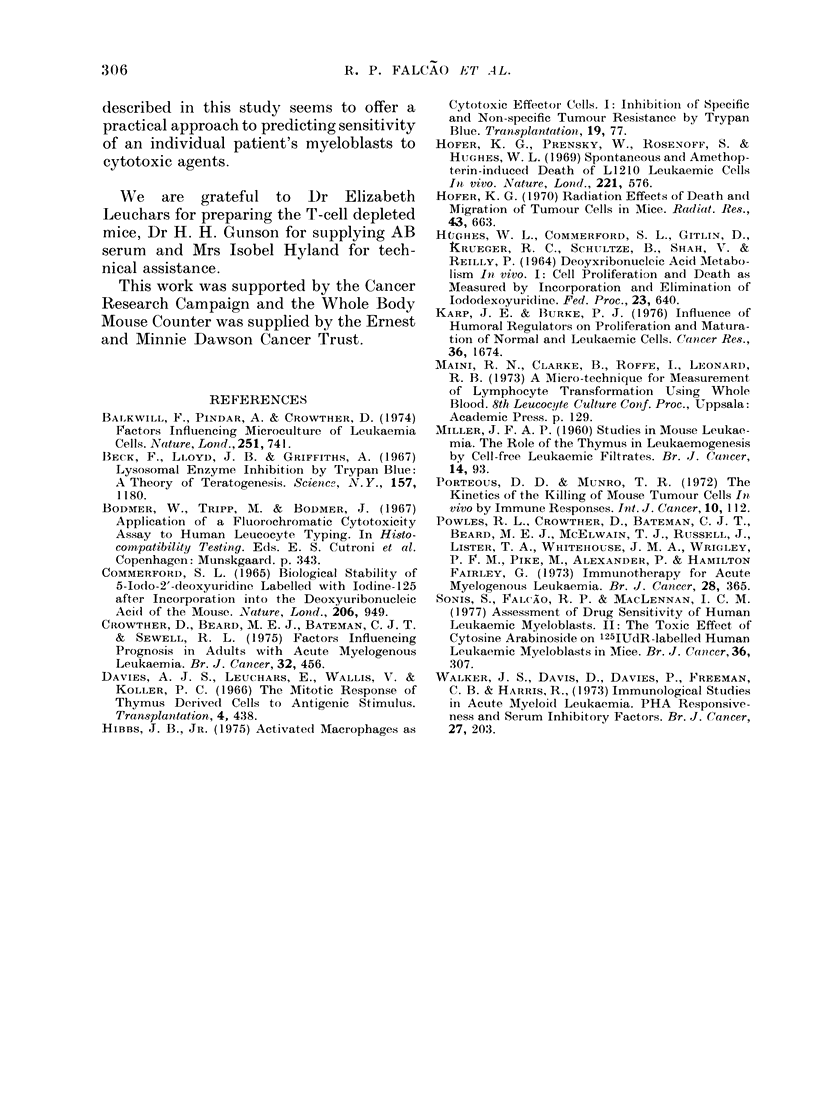# Assessment of Drug Sensitivity of Human Leukaemic Myeloblasts

**DOI:** 10.1038/bjc.1977.193

**Published:** 1977-09

**Authors:** R. P. FalcãO, S. Sonis, I. C. M. MacLennan, D. Chassoux, A. J. S. Davies, T. R. Munro

## Abstract

The compound ^125^IUdR can be incorporated in a stable form into the DNA of cells. The isotope is released if labelled cells or their progeny die. Consequently the rate of ^125^I excretion from mice can be used to follow the fate of labelled cells *in vivo.* Using these principles we show:

(1) Sufficient label can be incorporated *in vitro* into both fresh and cryopreserved human leukaemic myeloblasts, in non-toxic concentrations, to allow their survival in mice to be estimated by whole-body counting;

(2) The release of isotope from labelled cells is sufficiently slow to offer reasonable expectation that this technique can be used for assessing the sensitivity of myeloblasts to cytotoxic agents *in vivo* (an application described in the second paper in this series, Sonis, Falcão and MacLennon, 1977);

(3) The rate of ^125^Iexcretion from mice injected with myeloblasts from different donors varies. This probably reflects different rates of spontaneous death of injected myeloblasts;

(4) Active rejection of myeloblasts starts within 48 h of their injection into mice;

(5) Indirect evidence that phagocytic cells may be active agents in myeloblast destruction in mice;

(6) Various methods of immunologically depriving mice were assessed to see if they would result in a useful increase in survival of injected human myeloblasts. We conclude that there is little advantage and some limitations in using mice thus deprived;

(7) One of the agents used for immunological deprivation—silica powder—markedly decreased the rate of ^125^I loss from mice injected with labelled *killed* myeloblasts. This experience emphasizes the importance of including the killed-cell control in this assay.


*Br. J. Cancer* (1977) **36**, 297

### ASSESSMENT OF DRUG SENSITIVITY OF HUMAN LEUKAEMIC MYELOBLASTS

### I. LABELLING HUMAN MYELOBLASTS WITH $^{125}$IUdR FOR SURVIVAL STUDIES IN MICE

R. P. FALCÃO,* S. SONIS,† I. C. M. MacLENNAN, D. CHASSOUX,‡

A. J. S. DAVIES§ AND T. R. MUNRO**

*From the Nuffield Department of Clinical Medicine, Radcliffe Infirmary, Oxford OX2 6HE, §Institute of Cancer Research, Fulham Road, London SW3, and **Nuclear Physics Laboratory, Keble Road, Oxford*

Received 12 July 1976 Accepted 25 April 1977

**Summary.—The compound $^{125}$IUdR can be incorporated in a stable form into the DNA of cells. The isotope is released if labelled cells or their progeny die. Consequently the rate of $^{125}$I excretion from mice can be used to follow the fate of labelled cells *in vivo*. Using these principles we show:**

**(1) Sufficient label can be incorporated *in vitro* into both fresh and cryopreserved human leukaemic myeloblasts, in non-toxic concentrations, to allow their survival in mice to be estimated by whole-body counting;**

**(2) The release of isotope from labelled cells is sufficiently slow to offer reasonable expectation that this technique can be used for assessing the sensitivity of myeloblasts to cytotoxic agents *in vivo* (an application described in the second paper in this series, Sonis, Falcão and MacLennon, 1977);**

**(3) The rate of $^{125}$I excretion from mice injected with myeloblasts from different donors varies. This probably reflects different rates of spontaneous death of injected myeloblasts;**

**(4) Active rejection of myeloblasts starts within 48 h of their injection into mice;**

**(5) Indirect evidence that phagocytic cells may be active agents in myeloblast destruction in mice;**

**(6) Various methods of immunologically depriving mice were assessed to see if they would result in a useful increase in survival of injected human myeloblasts. We conclude that there is little advantage and some limitations in using mice thus deprived;**

**(7) One of the agents used for immunological deprivation—silica powder—markedly decreased the rate of $^{125}$I loss from mice injected with labelled *killed* myeloblasts. This experience emphasizes the importance of including the killed-cell control in this assay.**

THE overall remission rate in acute myeloblastic leukaemia in many centres is over 50%. However, the number of patients who survive the hazards of the first few weeks of treatment, but still fail to achieve remission because of lack of sensitivity to drugs, is disappointingly high.

With this in mind, we have set out to establish a technique which could be used to assess the sensitivity of individual patients' myeloblasts to various cyto-

* Supported by the Fundacão Amparo Pesquisa Estado de São Paulo.
Present address: Faculdade Medicina Ribeirao, Preto, Brasil.
† Present address: Department of Surgery, Peter Bent Brigham Hospital, 721 Huntington Avenue, Boston, Mass. 02115, U.S.A.
‡ Present address: Pasteur Institute, 59, Lille, France.

toxic agents *in vivo*. The model we describe labels patients' leukaemic myeloblasts *in vitro* with the thymidine analogue 5-$^{125}$Iodo-2′-deoxyuridine ($^{125}$IUdR). The leukaemic cells are then injected into mice, and their survival measured by release of isotope from leukaemic cells. This release is measured by whole-body mouse counting, using the techniques established by Hughes *et al.* (1964) and Commerford (1965). These workers showed that IUdR incorporated into DNA is only released from cells at death. Furthermore, re-utilisation of released isotope in mice given iodide in their drinking water to block thyroid uptake is minimal.

In this paper we report experiments which establish suitable labelling conditions for both fresh and cryopreserved human leukaemic myeloblasts. We then describe observations on the way in which these labelled cells are handled by normal and immunologically deprived mice. The second paper in this series shows how this technology can be applied to the study of drug sensitivity in individual patient's leukaemic cells (Sonis *et al.*, 1977).

### MATERIAL AND METHODS

*Myeloblasts.*—Myeloblasts were separated from patients with acute myeloblastic leukaemia (AML). Bone-marrow cells were collected into heparin (10 $\mu$l/ml) by sternal or iliac puncture. Red blood cells and neutrophils were then removed by centrifugation through a Ficoll-Triosil gradient of sp. gr. 1·080. Myeloblasts were isolated from venous blood by one-$g$ sedimentation. They were collected either by simple venesection or by a cell separator (Hemmenetic). The cells were washed once and either used immediately or aliquots containing $5 \times 10^8$ to $10^9$ cells in 1 ml of medium with 10% autologous plasma and 10% dimethylsulphoxide were frozen at 1 °C/min and stored over liquid $N_2$. We are grateful to Professors Beeson and Weatherall, and Drs Callender, Emmerson, Sharpe, Pippard and Bunch, for allowing us to study myeloblasts and serum from their patients.

Before use, the cells were rapidly thawed and diluted over 10 min to 20 ml in RPMI 1640 plus 10% foetal bovine serum (Gibco-Biocult) and washed twice. Samples used in this study comprised over 90% myeloblasts.

*Cultures.*—The medium consisted of RPMI 1640, with 20 u/ml of penicillin, 100 $\mu$g/ml of streptomycin and fresh glutamine. Different serum supplements were tested. $^{125}$IUdR at a specific activity of 25-35 Ci/mmol (Amersham) was utilised in all the experiments. The optimum concentration of the isotope was investigated and is reported in the results.

Incubations of myeloblasts and IUdR were carried out in glass medical flat bottles at 37 °C in an atmosphere of 95% air : 5% $CO_2$. Varying lengths of time, different volumes of medium and different cell densities were tested. At the end of the incubation period, the cells were flushed from the surface by injection of medium. They were centrifuged at 200 $g$ for 20 min and resuspended in 20 ml of fresh RPMI 1640 and centrifuged through 2 ml of foetal bovine serum (FBS) which had been layered under the cell suspension. This procedure removes free isotope, which remains above the FBS. The cells were then suspended in RPMI and the radioactivity measured in a $\gamma$ counter taking into account sample volume and well geometry.

*Animals and $^{125}$IUdR estimation.*—A breeding nucleus of CBA/H T6T6 strain originally from the M.R.C. Laboratory Animal Centre was maintained by brother–sister mating in the Animal House of the Nuffield Department of Clinical Medicine, Radcliffe Infirmary. Mice of either sex were used at 2–4 months of age.

In each experiment, groups of 4–5 animals of the same sex and age were utilised. Uptake of the iodine by the thyroid was avoided by giving 0·1% KI in the drinking water, 2 days before the labelled cells were injected, and for the duration of the experiment.

Three to five $\times 10^7$ cells in 1 ml of RPMI were injected i.p. and the radioactivity of the whole body was measured in a large NaI well-counter, immediately after injection and daily thereafter, using the technique of Hughes *et al.* (1964).

*Effect of serum on phytohaemagglutinin-stimulated blastogenesis.*—Fresh frozen serum from AML patients was tested for its ability to modify PHA-stimulated blastogenesis of normal human lymphocytes. The test was performed using a modification of the wholeblood assay described by Maini *et al.* (1973). One tenth of a ml of defibrinated whole blood, 0·1 ml of test serum, 0·7 ml of medium (Eagles basal medium with 1% antibiotics and 1% non-essential amino acids) and 0·1 ml of PHA at a 1 : 300 dilution (Wellcome Lab reagent grade) were added to (76 × 12 mm) polystyrene culture tubes. These cultures were incubated for 68 h at 37 °C in an atmosphere of 95% air and 5% $CO_2$. One $\mu$Ci of $^3$H-thymidine (Amersham) at 1 Ci/mmol in a volume of 0·05 ml was added, and the tubes were then incubated for 4 h at 37 °C. Cold acetic acid (2%) was then added and the mixture allowed to stand for 10 min. After centrifugation the cells were washed twice with 5% TCA at 4 °C and then washed once with cold methanol. The tubes were inverted and the pellets allowed to dry at room temperature. The dried pellets were then suspended in 0·3 ml of 0·1 N NaOH and 0·2 ml of the resulting suspension was removed and added to 10 ml of scintillation fluid. Samples were counted in a beta scintillation counter and allowance made for background and quenching. Each serum sample was tested in triplicate.

*Conditions for assessing the effect of AML sera on IUdR uptake by myeloblasts.*—Eighteen × $10^7$ myeloblasts were cultured for 20 h in 25ml universal vessels. The culture medium was RPMI with 10% of the serum supplement being assessed. At the end of the culture the cells were prepared for counting as indicated below.

*Harvesting cultures labelled with IUdR for counting* in vitro.—Such cultures were centrifuged and the pellet was resuspended in 5% trichloroacetic acid (TCA) at 4 °C. The precipitate was then centrifuged, washed in TCA at 4°C and recentrifuged. The supernatant was discarded and the residue counted in a Wallac well scintillation counter.

*Irradiated mice.*—Mice confined in a narrow perspex cage received 800 rad whole-body irradiation from a $^{60}$CO source at 45 rad/min. Animals were used 3 days after irradiation. The mortality due to irradiation alone was 30% in the first week (most deaths occur on Days 5 and 6).

*Thymectomised, irradiated, bone-marrow reconstituted (B) mice.*—Mice were thymectomised at 8 weeks of age following the technique of Miller (1960). Two weeks later they were given 850 rad whole-body irradiation from a 220 KV Westinghouse X-ray machine, HVL 0·4 mm Cu, focal distance 100 cm, dose rate 60 rad/min with no added filtration. Within 2–3 h, the mice were injected with 5 × $10^6$ syngeneic bone-marrow cells. These immunologically deprived mice (Davies *et al.*, 1966) were then left for a period of 30 days before being used.

*Trypan blue and silica treatment of animals.*—Trypan blue (BDH) was dialysed against distilled water and diluted in sterile saline to a concentration of 20 mg/ml. Each animal was injected i.p. with 5 mg and 1 mg at 24 and 3 h respectively before the injection of labelled myeloblasts. One-mg s.c. injections were then given at Days 1 and 3 after myeloblast transfer. This technique is based upon the work of Hibbs (1975).

All mice given a single injection of 10 mg of trypan blue died.

*Powdered silica.*—This was a generous gift from Dr L. Le Bouffant (CERCHAR, B.P.2, 60550 Verneuil-en-Hallatte, France). 85% of the granules were <1 $\mu$m diameter, 12% were 1–2 $\mu$m diameter and <1% were >3 $\mu$m diameter. The agent was given i.p. as a single injection of 20 mg 24 h before labelled myeloblasts.

*Preparation of peritoneal cells.*—Under brief ether anaesthesia the mice were injected i.p. with two 5-ml aliquots of RPMI plus heparin (10 $\mu$/ml). The free fluid in the peritoneal cavity was then drained into universal bottles (Sterilin). Nucleated cells were counted in an improved Neubauer counting chamber and all "smears" were prepared using a cytocentrifuge (Shandon). These preparations were fixed in methanol and stained with Giemsa.

*Killed cells.*—Labelled cells were killed by twice rapidly freezing (in liquid $N_2$) and thawing. The cells were then heated at 56 °C for 30 min.

### RESULTS

### (A) *Identification of suitable labelling conditions*

In this section we describe the experiments which led us to formulate a suitable set of conditions for labelling both fresh and cryopreserved myeloblasts. The criteria for successful labelling were: firstly, achieving sufficient counts incorporated into myeloblasts to enable external detection by whole-body counting when the cells were injected into mice; secondly, labelling in such a way as to cause minimum damage to the myeloblasts. Many of the variables assessed in this section had to be tested in parallel in large experiments, but they are dealt with here one by one for clarity.

(i) *Concentration of IUdR.*—Myeloblasts from a number of patients were set up in 20-h cultures with 10% FBS supplementing the standard medium. The cells were at a concentration of $10^6$ cells/ml and the medium was 1 cm deep in the culture bottles. Under the conditions using Amersham $^{125}$IUdR at sp. act. 25–35 Ci/mmol it was found that the radiochemical had to be used at 0·012 $\mu$Ci/ml or less to avoid detectable toxicity to the myeloblasts. The method for detection of toxicity was to assess the rate of $^{125}$I excretion from mice injected with myeloblasts labelled with IUdR. Fig. 1 shows a representative experiment, testing different concentrations of label. On the basis of these experiments, 0·006 $\mu$Ci/ml was chosen as a standard labelling concentration. There is no advantage in reducing the specific activity of label, as fewer counts are incorporated into myeloblasts before the threshold of toxicity is reached.

(ii) *Duration of labelling.*—This is an important variable. Using 0·006 $\mu$Ci/ml at the cell concentrations described above, it was found that, although as much label was taken up by the cells within 1·5 h as by 20 h, only a small proportion of the label taken up by 1·5 h was precipitable by 5% TCA at 4 °C. On the other hand, most of the label after 20 h was in DNA, as estimated by TCA precipitation (Fig. 2). Mice injected with myeloblasts labelled for 3·5 h were found to lose 65% of their IUdR (geometric mean of 5 animals) by the end of the first day after injection, while mice injected with myeloblasts labelled for 20 hours, only lost 18% of label during the first day. During the second and subsequent days, the logarithmic loss of label was the same in these two groups.

FIG. 1.—The loss of $^{125}$I from mice after i.p. injection of 5 × $10^7$ IUdR-labelled myeloblasts cultured for 20 h in: 0·006 $\mu$Ci/ml (● – – – ●); 0·02 $\mu$Ci/ml (○ ——— ○) or 0·03 $\mu$Ci/ml (□ – · – · – □). (■ . . . . ■) = myeloblasts labelled with 0·006 $\mu$Ci/ml and killed before injection. Each point is the geometric mean ± s.e. of 5 animals in each group.

FIG. 2.—Uptake of $^{125}$IUdR by myeloblasts cultured for varying lengths of time. The proportion of the total uptake precipitable by 5% TCA at 4 °C is shown by shaded areas. Vertical bars represent ± s.e., triplicate cultures.

(iii) *Concentration of cells.*—Two factors were considered here: firstly the concentration of cells/ml and secondly the depth of medium in the bottles. Using 20-h cultures with 0·006 $\mu$Ci/ml it was found that good labelling/cell was achieved at $5 \times 10^5$ or $10^6$ cells/ml where the depth of medium in the bottles was 1 cm. Greater cell concentrations resulted in less label being taken up per cell. This reduced uptake per cell could not be counteracted by increasing the isotope concentration, for such increases caused toxicity to myeloblasts.

(iv) *Serum supplement to medium.*—This was an important factor influencing labelling. Different results were obtained with different batches of the same type of serum. While some batches of foetal bovine serum were satisfactory, the best results were consistently obtained using 10% heat-inactivated pooled human AB serum. (We are grateful to Dr H. H. Gunson for gifts of AB serum.) Heparinised plasma was found to be as good as serum from the same source. Plasma or serum from AML patients was generally satisfactory as long as it was heat-inactivated. Some fresh AML sera markedly inhibited spontaneous uptake of IUdR by both autologous and allogeneic myeloblasts. Consequently we compared the uptake of IUdR by allogeneic myeloblasts, using fresh frozen and heat-inactivated sera from each of 53 untreated AML donors (Fig. 3). The results show that, overall, more IUdR is taken up by myeloblasts in heat-inactivated as opposed to fresh sera.

FIG. 3.—The effect on IUdR uptake by leukaemic myeloblasts from a single donor, of each of 53 sera from 53 untreated patients with AML, when the sera are used as a 10% supplement to culture medium. .... Fresh frozen serum; ——— heat activated serum.

The effect on [$^3$H]TdR uptake by lymphocytes from a single healthy donor of each of 34 fresh frozen sera from 34 untreated patients with AML, – – – –. The serum was used as a 10% supplement to medium which also contained PHA. [$^3$H]TdR was added to cultures between 68 and 72 h after the cultures were established.

The results are calculated as the uptake in an AML serum, as a % of the uptake in pooled AB serum, and plotted as a histogram. The groups on the horizontal axis are divided into 10 equal arithmetic groups where the uptake was less in AML than in AB serum and an eleventh group where the uptake was the same or greater than in AB serum.

Walker *et al.* (1973) described an inhibitory effect of sera from patients with AML on the uptake of IUdR by lymphocytes transformed with PHA. We, therefore, compared the effect of fresh frozen serum from each of 34 untreated AML donors on the uptake of IUdR by allogeneic myeloblasts, with their effect on PHA-induced blastogenesis of allogeneic lymphocytes from normal donors (Fig. 3). The experimental conditions are described in the Methods. While very poor [$^3$H]-TdR uptake by lymphocytes cultured with PHA occurred in some sera, there was no correlation between these sera and those associated with poor IUdR uptake by myeloblasts. All the sera used were presentation sera from patients who were subsequently treated with the Barts III regimen of chemotherapy for induction of remission (Powles *et al.*, 1973). We therefore examined the data for correlation between the degree of these serum effects and the chance of a serum donor achieving remission. No correlation was found in either system, using both the

Wilcoxon sum of ranks test comparing remitters and non-remitters, or a $\chi^2$ test in which patients were grouped as inhibitors (*i.e.* greater than 20% inhibition compared with AB serum pool) and non-inhibitors, remitters and non-remitters.

(B) *The incorporation of IUdR by fresh* versus *cryopreserved myeloblasts*

Cells were labelled with 0·006 $\mu$Ci/ml $^{125}$IUdR for 20 h at the concentration of cells described above. Viability of the stored cells was good, whether assessed by fluorescein diacetate uptake and conversion or trypan blue exclusion. Although the rate of uptake of isotope was reduced between 3 and 7-fold after cryopreservation (Table), the rate of release of isotope in mice from cryopreserved cells was similar to that seen from fresh cells.

(C) *Investigation of factors affecting the survival of myeloblasts in mice and attempts to prolong myeloblast survival*

In these experiments myeloblasts were labelled with 0·006 $\mu$Ci/mmol IUdR for 20 h. Cells were at a concentration of $10^6$/ml and the medium was 1 cm deep.

(i) *The cellular composition of mouse peritoneal fluid following the i.p. injection of labelled myeloblasts.*—Fifteen mice were injected with myeloblasts from a single patient. Five of the mice received IUdR-labelled cells. This group was counted each day for $^{125}$I retention. Two of the mice given unlabelled cells was sacrificed immediately after injection and further pairs were killed at 24 h intervals thereafter. The peritoneal fluid from these mice was washed out with medium. The cells were counted and a differential count was performed on a Giemsa-stained film. The cytological features of the myeloblasts allowed them to be clearly distinguished from mouse peritoneal cells. Host cells were classified as lymphocytes, macrophages and neutrophils, on conventional morphological grounds.

TABLE.—*Incorporation of $^{125}$IUdR by $10^7$ Frozen or Fresh Myeloblasts*

| Patient | Counts/min/$10^7$ cells | | Viability of Frozen Cells | |
|---|---|---|---|---|
| | Fresh | Frozen | % Trypan blue | % FDA + * |
| A | 903 | 432 | 93 | 90 |
| B | 1440 | 225 | | |
| C | | 630 | 95 | 90 |
| D | 1000 (1500) | 234 | 92 | 95 |
| E | 1500 (3200) | 270 | 86 | |
| F | 600 | 288 | 90 | 90 |
| G | | 108 | 93 | |
| H | | 540 | 90 | |
| I | | 90 | 85 | |
| J | | 216 | | |
| K | 54 | | | |
| L | | 342 | 92 | |
| M | 500 | | | |

(In brackets, bone marrow cells).

Trypan blue uptake was assessed by incubating in RPMI with 10% AB serum and 0·25% trypan blue at 37 °C for 5 min.

* Fluorescein diacetate uptake and conversion carried out according to Bodmer, Tripp and Bodmer (1967).

FIG. 4.—Peritoneal cavity cells after the injection of 5 × $10^7$ myeloblasts (○——○) macrophages, (● – – – ●) myeloblasts, (□ – · – · – □) lymphocytes and (■ . . . ■) polymorphonuclear cells. Each point is the mean of two animals. (▲ – · – · – ▲) = the release of $^{125}$I from 5 × $10^7$ IUdR-labelled myeloblasts (geometric mean of 5 mice). Day 1 is 24 h after injection of myeloblasts. The mean peritoneal exudate content of non-injected CBA mice = neutrophils <0·1 × $10^6$; lymphocytes, 0·7 × $10^6$; and macrophages, 0·2 × $10^6$.

The absolute counts of different cell types, together with the geometric mean of the percent $^{125}$I retention, are shown in Fig. 4. There was a rapid but transient increase in neutrophil numbers, so that they were the most abundant host cell at 24 h. By 48 h, macrophages had become the dominant host cell and there was already a marked diminution of myeloblasts. At this stage, frequent myeloblast macrophage interactions were seen, including phagocytosis of myeloblasts. The discrepancy between $^{125}$I counts retained and peritoneal myeloblast content, at this stage, leads one to postulate that either some myeloblasts have left the peritoneal cavity, or some are firmly adherent to the wall of the peritoneum. Experiments with injected killed myeloblasts (Fig. 5) makes unlikely the explanation that $^{125}$I is being retained for a significant period after myeloblast death. This experiment was repeated once, with similar results.

(ii) *Evidence for active host participation in causing myeloblast death.*—The first series of experiments designed to answer this question involved priming mice with an i.p. injection of unlabelled myeloblasts 2 days before giving labelled cells. Fig. 5 shows the results of one such experiment, where $^{125}$I excretion is compared in primed and unprimed mice. It will be seen that the pre-exposure to myeloblasts results in a 24-h advance of the slope of $^{125}$I excretion. This experiment is highly reproducible, and myeloblasts from a different donor are equally effective at inducing accelerated rejection. Although these data do not give any information about the specificity of this reaction they do indicate host involvement in the destruction of myeloblasts. The next series of experiments was designed to see whether pretreatment of mice with agents which would suppress various types of cytotoxic immunity could delay myeloblast rejection.

FIG. 5.—Release of $^{125}$I after i.p. injection of $5 \times 10^7$ IUdR-labelled myeloblasts into mice. Normal (● – – –●) and primed (○———○) with $5 \times 10^7$ non-labelled cells 48 h earlier. Geometric mean ± s.e. of 5 animals.

(iii) *The effect of macrophage blocking on myeloblast rejection.*—In preliminary experiments, macrophage blocking was carried out using silica powder injected i.p. This agent considerably delayed the rate of $^{125}$I loss from animals injected with viable myeloblasts. However, it also markedly reduced the rate of clearance of $^{125}$I from mice given killed cells. Consequently, for this practical reason, silica was not considered suitable for use as an agent to delay myeloblast rejection.

The second macrophage-blocking agent to be used was trypan blue. This was found by Beck, Lloyd and Griffiths (1967) to block lysosome activity, and Hibbs (1975) used this agent to inhibit tumour-cell damage by macrophages. Experiments using this agent, alone and in combination with whole-body irradiation, are shown in Figure 6. In otherwise normal mice, trypan blue had a marginal slowing effect on myeloblast rejection. However, if mice were given 800 rad 3 days before injection of myeloblasts, and were also given trypan blue, $^{125}$I excretion was markedly retarded. This retardation was not associated with loss of ability to excrete $^{125}$I from dead cells. Irradiation

FIG. 6.—Release of $^{125}$I after i.p. injection of 3 × 10$^7$ live IUdR-labelled myeloblasts in normal mice (■ . . . ■), trypan-blue treated (▲ – · – · – ▲) (injected i.p. with 5 mg and 1 mg, 24 and 3 h before the injection of labelled cells and 1 mg s.c. on Days 1 and 3), irradiated (○———○) (mice given 800 rad 3 days before injection of labelled cells) and irradiated + trypan blue (● – – – – ●) mice. Mean ± s.e. of 4 animals.

3 × 10$^7$ killed IUdR-labelled myeloblasts were injected into animals prepared as for each of the four different groups. The results for all these animals were essentially the same, and are plotted as a single line (□ – · – · – □).

FIG. 7.—Release of $^{125}$I after injection of: 5 × 10$^7$ IUdR-labelled live myeloblasts in normal mice i.p. (○ ——— ○) and s.c. (● – – – – ●); 5 × 10$^7$ IUdR-labelled killed myeloblasts in normal mice injected i.p. (□ – · – · – □) and s.c. (■ . . . . ■). Means ± s.e. for 5 animals.

alone only marginally reduced the speed of myeloblast rejection; i.e., both irradiation and trypan blue were required to produce a significant prolongation of survival of myeloblasts in mice.

(iv) *The effect of T-cell depletion on myeloblast rejection.*—Mice were deprived of thymus-processed cells as is described in the Methods. These B mice were tested in parallel with controls for their capacity to reject labelled human leukaemic myeloblasts.

There was a decrease in the rate of rejection of about 12–24 h in the B mice compared with controls. This delay was not increased further by trypan blue treatment.

(v) *Effect of s.c. injection of myeloblasts on their survival.*—The trypan blue and cytological studies suggested that some active toxic effect is being expressed against myeloblasts by macrophages. We, therefore, set up experiments to see whether labelled myeloblasts injected s.c. where there is not a high natural macrophage population, lost $^{125}$I more slowly than those injected i.p. Fig. 7 shows a representative experiment indicating that s.c. myeloblasts do indeed survive longer than the same cells injected i.p.

### DISCUSSION

The response of mouse tumour cells to chemotherapy, irradiation and host immunity has been studied by a number of workers using the IUdR labelling technique (Hofer *et al.*, 1969; Hofer, 1970; Porteous and Munro, 1972). Human leukaemic myeloblasts, however, require rather different labelling conditions from those used for serially transplanted tumours, which have a uniformly high labelling index. Short pulses with relatively high concentration of IUdR are both toxic and also give poor labelling of myeloblasts. By the use of low concentrations of this radiochemical over a prolonged incubation period, we have shown that it is possible to label these cells from most patients with sufficient counts to permit cell-survival studies to be carried out in mice. The toxicity and spontaneous release of $^{125}$IUdR used in this way compares very favourably with most pre-labelling techniques used for cytotoxicity testing *in vitro*. Consequently, we have progressed to evaluation of the clinical usefulness of this technique in assessing drug sensitivity in AML with some optimism. The second paper in this series (Sonis *et al.*, 1977) reports that, in a limited study, the assay may be able to predict sensitivity to cytosine arabinoside.

The low labelling index of myeloblasts (Crowther *et al.*, 1975) does mean that only a relatively small sample of leukaemic cells is being labelled. It can be argued that this actively dividing fraction may not be representative of all the neoplastic cells. The only answer to this is practical evaluation, and the point is considered further in the next paper. In any case, the labelling procedure used here is likely to be at least as representative of the total neoplasm as cells obtained following prolonged culture *in vitro* or serial transplantation in immunologically deprived animals.

The main advantages of using an *in vivo* test system, even if it does cross a species barrier, is that many cytotoxic agents, for example cyclophosphamide and azathioprine, are broken down to active daughter compounds *in vivo*. Obviously, the model we propose cannot predict an individual's capacity to break down a drug outside neoplastic cells.

There can be little doubt that much of the $^{125}$I excretion observed in our studies is due to spontaneous death of myeloblasts. However, there does appear to be some reaction by the mouse to the myeloblasts which accelerates loss of $^{125}$I following the injection of viable cells. This is shown by the consistent finding that the main slope of $^{125}$I excretion occurred earlier if unlabelled myeloblasts were injected 2 days before the labelled myeloblasts. This cannot just be an effect on release and excretion of iodide from dead cells, as the priming had no effect on the rate of excretion of $^{125}$I from mice injected with *dead* labelled cells. The mechanism involved here is unclear, although a number of the experiments provide an indirect hint that macrophages may be involved. The important conclusion to be drawn from the immunological deprivation experiments is that the techniques of deprivation used do not delay $^{125}$I release sufficiently to be of practical advantage. Indeed, as they are somewhat drastic, they might alter the recipient's capacity to handle cytotoxic agents. In this context, the marked effect of silica on the excretion of iodide following injection of dead labelled cells is a warning that the killed-cell control must be included in all drug evaluation studies.

The growth-inhibiting factors which we describe in this paper have been recognised previously. Balkwill, Pindar and Crowther (1974) found a "complement"-dependent growth-inhibitory factor in a patient with AML. Karp and Burke (1976) have also described an inhibitory effect of this type in leukaemic patients' serum, which acted on both normal and leukaemic bone marrow cells.

Walker *et al.* (1973) described an inhibitory effect of sera from patients with AML on the mitotic response of normal lymphocytes to PHA. It is disappointing that no correlation of these factors with remission achievement was apparent.

In conclusion we feel that the incidence of failure to achieve remission through lack of response to drugs is sufficiently high to justify the development of predictive tests for drug sensitivity in acute myeloblastic leukaemia. The technique described in this study seems to offer a practical approach to predicting sensitivity of an individual patient's myeloblasts to cytotoxic agents.

We are grateful to Dr Elizabeth Leuchars for preparing the T-cell depleted mice, Dr H. H. Gunson for supplying AB serum and Mrs Isobel Hyland for technical assistance.

This work was supported by the Cancer Research Campaign and the Whole Body Mouse Counter was supplied by the Ernest and Minnie Dawson Cancer Trust.

### REFERENCES

BALKWILL, F., PINDAR, A. & CROWTHER, D. (1974) Factors Influencing Microculture of Leukaemia Cells. *Nature, Lond.*, **251**, 741.

BECK, F., LLOYD, J. B. & GRIFFITHS, A. (1967) Lysosomal Enzyme Inhibition by Trypan Blue: A Theory of Teratogenesis. *Science, N.Y.*, **157**, 1180.

BODMER, W., TRIPP, M. & BODMER, J. (1967) Application of a Fluorochromatic Cytotoxicity Assay to Human Leucocyte Typing. In *Histocompatibility Testing.* Eds. E. S. Cutroni *et al.* Copenhagen: Munskgaard. p. 343.

COMMERFORD, S. L. (1965) Biological Stability of 5-Iodo-2′-deoxyuridine Labelled with Iodine-125 after Incorporation into the Deoxyuribonucleic Acid of the Mouse. *Nature, Lond.*, **206**, 949.

CROWTHER, D., BEARD, M. E. J., BATEMAN, C. J. T. & SEWELL, R. L. (1975) Factors Influencing Prognosis in Adults with Acute Myelogenous Leukaemia. *Br. J. Cancer*, **32**, 456.

DAVIES, A. J. S., LEUCHARS, E., WALLIS, V. & KOLLER, P. C. (1966) The Mitotic Response of Thymus Derived Cells to Antigenic Stimulus. *Transplantation*, **4**, 438.

HIBBS, J. B., JR. (1975) Activated Macrophages as Cytotoxic Effector Cells. I: Inhibition of Specific and Non-specific Tumour Resistance by Trypan Blue. *Transplantation*, **19**, 77.

HOFER, K. G., PRENSKY, W., ROSENOFF, S. & HUGHES, W. L. (1969) Spontaneous and Amethopterin-induced Death of L1210 Leukaemic Cells *In vivo. Nature, Lond.*, **221**, 576.

HOFER, K. G. (1970) Radiation Effects of Death and Migration of Tumour Cells in Mice. *Radiat. Res.*, **43**, 663.

HUGHES, W. L., COMMERFORD, S. L., GITLIN, D., KRUEGER, R. C., SCHULTZE, B., SHAH, V. & REILLY, P. (1964) Deoyxribonucleic Acid Metabolism *In vivo.* I: Cell Proliferation and Death as Measured by Incorporation and Elimination of Iododexoyuridine. *Fed. Proc.*, **23**, 640.

KARP, J. E. & BURKE, P. J. (1976) Influence of Humoral Regulators on Proliferation and Maturation of Normal and Leukaemic Cells. *Cancer Res.*, **36**, 1674.

MAINI, R. N., CLARKE, B., ROFFE, I., LEONARD, R. B. (1973) A Micro-technique for Measurement of Lymphocyte Transformation Using Whole Blood. *8th Leucocyte Culture Conf. Proc.*, Uppsala: Academic Press. p. 129.

MILLER, J. F. A. P. (1960) Studies in Mouse Leukaemia. The Role of the Thymus in Leukaemogenesis by Cell-free Leukaemic Filtrates. *Br. J. Cancer*, **14**, 93.

PORTEOUS, D. D. & MUNRO, T. R. (1972) The Kinetics of the Killing of Mouse Tumour Cells *In vivo* by Immune Responses. *Int. J. Cancer*, **10**, 112.

POWLES, R. L., CROWTHER, D., BATEMAN, C. J. T., BEARD, M. E. J., MCELWAIN, T. J., RUSSELL, J., LISTER, T. A., WHITEHOUSE, J. M. A., WRIGLEY, P. F. M., PIKE, M., ALEXANDER, P. & HAMILTON FAIRLEY, G. (1973) Immunotherapy for Acute Myelogenous Leukaemia. *Br. J. Cancer*, **28**, 365.

SONIS, S., FALCÃO, R. P. & MACLENNAN, I. C. M. (1977) Assessment of Drug Sensitivity of Human Leukaemic Myeloblasts. II: The Toxic Effect of Cytosine Arabinoside on $^{125}$IUdR-labelled Human Leukaemic Myeloblasts in Mice. *Br. J. Cancer*, **36**, 307.

WALKER, J. S., DAVIS, D., DAVIES, P., FREEMAN, C. B. & HARRIS, R., (1973) Immunological Studies in Acute Myeloid Leukaemia. PHA Responsiveness and Serum Inhibitory Factors. *Br. J. Cancer*, **27**, 203.